# Combined analysis of dissimilar promoter accessibility and gene expression profiles identifies tissue-specific genes and actively repressed networks

**DOI:** 10.1186/s13072-019-0260-2

**Published:** 2019-02-22

**Authors:** Rebekah R. Starks, Anilisa Biswas, Ashish Jain, Geetu Tuteja

**Affiliations:** 1 Genetics, Development, and Cell Biology, Iowa State University, Ames, IA 50011 USA; 2 Bioinformatics and Computational Biology, Iowa State University, Ames, IA 50011 USA; 3 Molecular, Cellular, and Developmental Biology, Iowa State University, Ames, IA 50011 USA

**Keywords:** ATAC-seq, Gene regulation, RNA-seq, Promoter coverage, Tissue-specific genes, Gene networks

## Abstract

**Background:**

The assay for transposase-accessible chromatin (ATAC-seq) is a powerful method to examine chromatin accessibility. While many studies have reported a positive correlation between gene expression and promoter accessibility, few have investigated the genes that deviate from this trend. In this study, we aimed to understand the relationship between gene expression and promoter accessibility in multiple cell types while also identifying gene regulatory networks in the placenta, an understudied organ that is critical for a successful pregnancy.

**Results:**

We started by assaying the open chromatin landscape in the mid-gestation placenta, when the fetal vasculature has started developing. After incorporating transcriptomic data generated in the placenta at the same time point, we grouped genes based on their expression levels and ATAC-seq promoter coverage. We found that the genes with the strongest correlation (high expression and high coverage) are likely involved in housekeeping functions, whereas tissue-specific genes were highly expressed and had only medium–low coverage. We also predicted that genes with medium–low expression and high promoter coverage were actively repressed. Within this group, we extracted a protein–protein interaction network enriched for neuronal functions, likely preventing the cells from adopting a neuronal fate. We further confirmed that a repressive histone mark is bound to the promoters of genes in this network. Finally, we ran our pipeline using ATAC-seq and RNA-seq data generated in ten additional cell types. We again found that genes with the strongest correlation are enriched for housekeeping functions and that genes with medium–low promoter coverage and high expression are more likely to be tissue-specific. These results demonstrate that only two data types, both of which require relatively low starting material to generate and are becoming more commonly available, can be integrated to understand multiple aspects of gene regulation.

**Conclusions:**

Within the placenta, we identified an active placenta-specific gene network as well as a repressed neuronal network. Beyond the placenta, we demonstrate that ATAC-seq data and RNA-seq data can be integrated to identify tissue-specific genes and actively repressed gene networks in multiple cell types.

**Electronic supplementary material:**

The online version of this article (10.1186/s13072-019-0260-2) contains supplementary material, which is available to authorized users.

## Background

The placenta is a transient organ, critical for fetal survival within the uterine environment. It is the only physical connection between the mother and fetus, supplying the fetus with the nutrients, oxygen, and hormones necessary for proper development [1, 2]. Rodents and primates have a hemochorial placenta [3], in which maternal blood is in direct contact with the chorion in the intervillous space. To allow for a more efficient exchange of oxygen and nutrients between maternal and fetal blood, the fetal placenta undergoes branching morphogenesis, which increases its surface area and allows a complex vascular network to develop [4]. In mice, defects in branching morphogenesis or vasculature development, sometimes associated with abnormal gene expression, commonly lead to embryonic lethality midway through gestation [1]. For example, simultaneous deletion of the Hey1 and Hey2 transcription factors results in embryonic lethality at approximately embryonic day (e) 9.5, due to decreased vascular remodeling [5]. As another example, EpCAM-null mice show evidence of insufficient vasculogenesis in the fetal placenta by e9.5, which contributes to embryonic lethality by e12.5 [6].

In addition to gene knockout studies, transcriptome studies have been carried out to identify genes that are expressed in the placenta at e9.5, as well as at other time points [7–9]. However, transcriptome studies alone cannot provide insight into tissue-specific gene networks when they are carried out in a single tissue, and also cannot provide information on genes that are actively repressed in a particular context. To better understand these aspects of gene regulation, chromatin immunoprecipitation followed by sequencing (ChIP-seq) data for several histone modifications, such as H3K4me3, H3K27ac [10], and H3K27me3 has been integrated with RNA-seq data across multiple tissues [11]. However, generating multiple ChIP-seq datasets with sufficient biological replicates and controls is costly and typically requires a large amount of starting material.

The assay for transposase-accessible chromatin using sequencing (ATAC-seq) is a promising recent technique that can be used to address some of these issues, requiring a relatively low amount of starting material [12]. Additionally, since ATAC-seq data are associated with nucleosome depletion, it can be used to identify genomic regions associated with gene activation or gene repression. Several studies that have integrated ATAC-seq and RNA-seq data reported a positive correlation between the ATAC-seq signal at a gene’s promoter and its expression [13–15]. Despite this correlation, many studies find that changes in the chromatin landscape are not always associated with expected changes in transcription [16–18]. Still, a thorough investigation into this correlation, and the genes that seem to deviate from it, is lacking.

In order to better understand gene regulation in the placenta, as well as the relationship between accessibility at a gene promoter and gene expression, we generated ATAC-seq data in the mouse placenta and integrated it with RNA-seq data generated in the same context [9]. We then defined gene groups based on the level of ATAC-seq signal at gene promoters and the corresponding gene expression level, according to RNA-seq. In addition to identifying genes with a strong positive correlation, we also identified genes with medium–low expression and high ATAC-seq promoter coverage, and genes with high expression and medium–low ATAC-seq promoter coverage. Investigating these gene groups further led us to identify a neuronal network that we predict is actively repressed in the placenta, as well as a set of placenta-specific genes and subnetworks that are associated with specific functions of the placenta. Finally, we analyzed previously published ATAC-seq and RNA-seq datasets generated in 10 additional contexts using our pipeline. We were consistently able to identify tissue-specific genes, as well as actively repressed gene networks. Therefore, in addition to identifying novel regulatory mechanisms in the placenta, we also better defined the relationship between promoter accessibility and gene expression and demonstrated how to integrate ATAC-seq and RNA-seq to identify tissue-specific genes.

## Results

### Identifying accessible regions in the mid-gestation mouse placenta

**Fig. 1:**
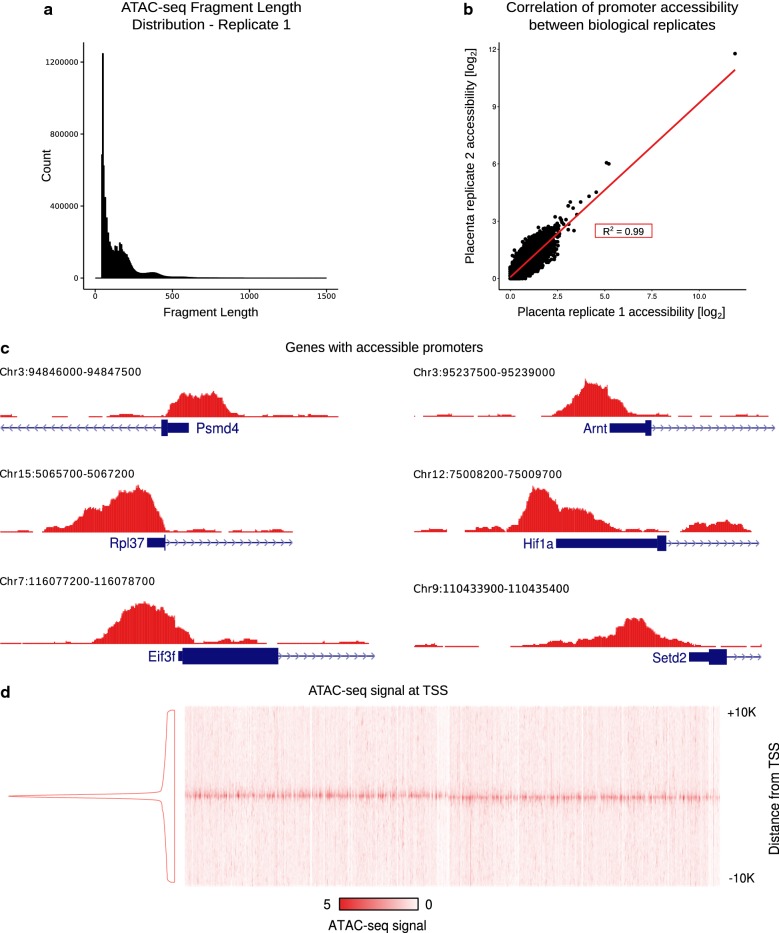
ATAC-seq analysis of e9.5 mouse placenta. **a** Distribution of ATAC-seq data fragment lengths from a representative sample (biological replicate 1). Small fragments correspond to open chromatin while peaks at larger fragment sizes represent fragments that span one or more nucleosomes. **b** Scatter plot representing promoter accessibility for each gene in replicate 1 plotted against the promoter accessibility for the corresponding gene in replicate 2. Biological replicates show a strong correlation, indicated in the red box (Pearson Correlation Coefficient). **c** Representative genes with a pileup of ATAC-seq reads in the promoter region. **d** Heatmap (total number of reads at the start site normalized by library size) and density plot of ATAC-seq fragments showing enrichment at gene TSSs (plot includes all knownGenes from the UCSC genome browser)

To define the chromatin landscape in mouse placenta at e9.5, we carried out ATAC-seq for three biological replicates, as described in the methods. Because ATAC-seq is infrequently carried out in whole tissue, we first assessed data quality. The fragment length distributions showed the expected periodicity before and after sequencing, with an abundance of fragments falling within nucleosome-free regions [≤ 140 base pairs (bp)] (Fig. 1a; Additional file 1: Figure S1a–c). We then aligned the reads to the mouse genome and calculated the coverage in gene promoters, defined as 500 bp surrounding the transcription start site (TSS). We found a high correlation between biological replicates [*R*^2^ = 0.99 (Pearson)] (Fig. 1b; Additional file 1: Figure S1d, e) and therefore combined reads across replicates for further analysis.

As expected, we observed a pileup of ATAC-seq reads (a peak) in the promoters of genes that are expressed in the placenta (Fig. 1c). A peak was observed in the promoters of many housekeeping genes, including Psmd4, Rpl37, and Eif3f, [19] as well as in the promoters of genes with known regulatory roles in multiple tissues including the placenta, such as Arnt [20], Hif1a [21], and Setd2 [22]. Finally, we found that in general, there is a strong ATAC-seq signal centered at gene TSSs (Fig. 1d). Together, these data demonstrate a high signal-to-noise ratio in the ATAC-seq data generated from whole mouse placenta.

### ATAC-seq signal at promoters is correlated with gene expression

**Fig. 2:**
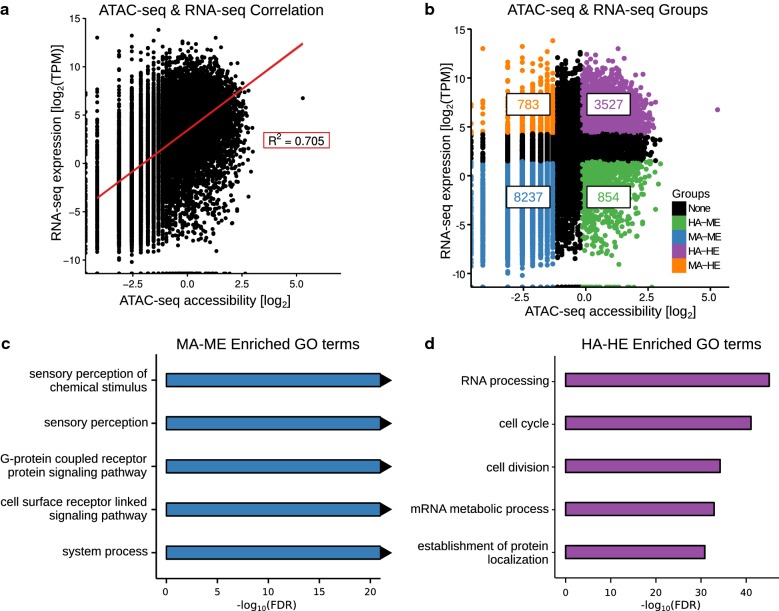
Promoter accessibility is strongly correlated with gene expression. **a** Scatter plot showing a strong positive correlation between promoter accessibility and gene expression. The correlation coefficient is shown in the red box (Spearman). **b** Genes are grouped based on the level of promoter accessibility and gene expression. The number of genes in each group is shown in the boxes. **c** Genes in the MA–ME group are enriched for biological processes related to sensory perception. The triangle at the end of the bar represents an FDR of 0. **d** Genes in the HA–HE group are enriched for biological processes related to housekeeping functions

Previous studies have reported a significant correlation between gene expression and the ATAC-seq signal at a gene’s promoter (the promoter accessibility) [13–15]. Therefore, we calculated the correlation of promoter accessibility with gene expression. Gene expression was measured using transcripts per million (TPM), calculated from previously published e9.5 placenta RNA-seq data [9]. In agreement with other studies, we found a strong correlation between accessibility and expression [*R*^2^ = 0.705 (Spearman); *p* value < 2.2e−16] (Fig. 2a). It is likely that a higher correlation is typically not observed because accessible regions are not always associated with gene activity. They can also be associated with gene repression or genes that are poised to become active [23–25]. Although some aspects of this correlation have been investigated, the majority of studies have not fully explored the relationship between ATAC-seq and RNA-seq data, especially with respect to genes that have low accessibility and a high level of expression. Therefore, to further understand the relationship between ATAC-seq and RNA-seq, we divided genes into groups based on their level of expression and promoter accessibility (see “Methods”). We found that the majority of genes (8237) had medium–low accessibility and medium–low expression (MA–ME), and the second largest group (3527 genes) had high accessibility and high expression (HA–HE) (Fig. 2b). To determine the biological functions associated with these groups, we carried out a functional enrichment analysis using the Genomic Regions Enrichment of Annotation Tool (GREAT) [26]. As expected, we found clear distinctions between the biological processes enriched in each group. For example, MA–ME genes are strongly enriched for terms related to sensory perception (Fig. 2c), whereas HA–HE genes are enriched for general cell functionality terms such as “cell cycle” and “RNA processing” (Fig. 2d). These findings are in agreement with previous studies. One such study, carried out in human T-helper cells, found that genes with accessible promoters and high expression were enriched for housekeeping functions, whereas those with inaccessible promoters were enriched for olfactory terms [27]. A more recent study also found that genes with accessible promoters in three different types of hematopoietic stem cells (HSCs) were enriched for terms related to regulating the cell cycle and DNA damage and repair [28].

We next carried out binding site enrichment analysis on the promoters of genes in the MA–ME and HA–HE groups, using promoters of genes in the other three groups as background. We identified multiple enriched motifs in the MA–ME group (Additional file 2: Table S1), including those for Pou2f1 and Foxj2, which are associated with olfactory gene regulation and development [29, 30]. Many more motifs were enriched in the HA–HE group (Additional file 2: Table S1), including several E2F and Ets motifs, which were previously found to be enriched in the promoters of housekeeping genes [31, 32].

Based on the enrichment of general cellular functionality terms and motif enrichment results, we predicted that a large number of housekeeping genes were in the HA–HE group. We therefore determined the percent overlap of MA–ME and HA–HE gene groups with 4781 housekeeping genes identified across 17 tissues [33]. We found that 57% of the HA–HE group were annotated as housekeeping, while only 0.2% of the MA–ME group were annotated as housekeeping genes (Additional file 1: Figure S2).

In addition to MA–ME genes and HA–HE genes, we found 783 genes that had medium–low accessibility and high expression (MA–HE), and 854 genes that had high accessibility and medium–low expression (HA–ME) (Fig. 2b). Because HA–ME and MA–HE genes are generally less understood, we further investigated how these two groups contribute to gene regulation in the placenta.

### HA–ME genes reveal a potentially repressed neuronal network

**Fig. 3:**
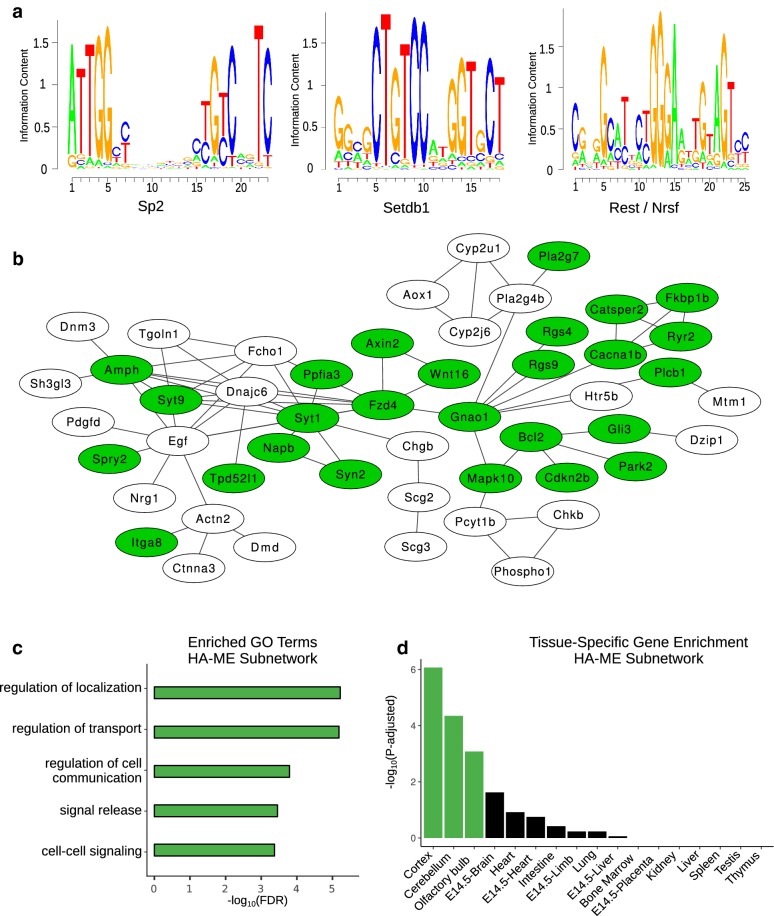
Analysis of HA–ME genes. **a** Motifs that are enriched in HA–ME gene promoters compared to background gene promoters in e9.5 placenta. **b** The largest PPI network in the HA–ME group, identified using STRING. Green nodes correspond to genes associated with the GREAT terms shown in **c**. **c** Bar plot of the enriched GO biological process terms for the subnetwork from **b**. Enriched terms are related to neuronal functions. **d** Tissue-specific gene enrichment analysis for genes in the subnetwork presented in **b** showing enrichment for genes expressed in brain-related tissues. Green colored bars correspond to tissues with an adjusted *p* value ≤ 0.01

Previous studies have identified regions of high accessibility that are also marked by a histone modification associated with gene repression, H3K27me3 [23, 34]. Therefore, we hypothesized that HA–ME genes may be actively repressed in the placenta. To identify potential transcription factors that may repress HA–ME genes, we scored motifs for transcription factors expressed in e9.5 placenta in HA–ME promoters using the PRISM [35] pipeline (see “Methods”). Three motifs were significantly enriched in HA–ME gene promoters relative to a background set of all gene promoters from each of the other three groups (Fig. 3a; Additional file 2: Tables S1, S2). The first enriched motif was for Sp2, a transcription factor that can repress cholesterol synthesis genes [36] and can form a repression complex with Klf6 to repress Mmp9 [37]. The second enriched motif was for Setdb1, a histone methyltransferase that trimethylates lysine 9 on histone H3. Setdb1 is known to interact with Oct4 in embryonic stem cells (ESCs) to repress genes involved in differentiation [38, 39]. The third enriched motif was for the Rest/Nrsf transcription factor. Rest/Nrsf is well known to be involved in preventing neuronal differentiation and function in nonneuronal tissues [40–42]. To further investigate potential repression networks in HA–ME genes, we used the STRING database [43] (see “Methods”) and identified a large protein–protein interaction (PPI) subnetwork of 50 genes (Fig. 3b). Interestingly, the most highly connected genes in this subnetwork were Fzd4, Egf, and Syt1, of which Fzd4 and Egf are each known to be involved in neuronal differentiation [44–47]. To confirm the potential repression of genes in this network, we performed ChIP-qPCR for H3K27me3 on nine target genes from the network. We included genes that have a known role in neuronal differentiation, as well as those that have no known role in this process. All nine targets were bound by H3K27me3 (fold-change ≥ 1.5) and five were strongly bound (fold-change ≥ 10), indicating the genes are in a repressed state (Additional file 1: Figure S3a). Next, we performed GO analysis of this network, which showed strong enrichment for terms such as “regulation of cell communication” and “cell–cell signaling” (Fig. 3c), which could be related to neuronal function. Using a randomization analysis (see “Methods”), we determined that these terms are more significant in the subnetwork than in random sets of 50 HA–ME genes (Additional file 1: Figure S3b–d).

Finally, we used TissueEnrich [48] to determine whether the subnetwork was enriched for tissue-specific genes. We found the strongest enrichment for brain tissues, including the cortex and cerebellum (Fig. 3d). Together, these data provide strong evidence that a neuronal network is actively repressed in the placenta.

### Placenta-specific genes have medium–low promoter accessibility and high expression

**Fig. 4:**
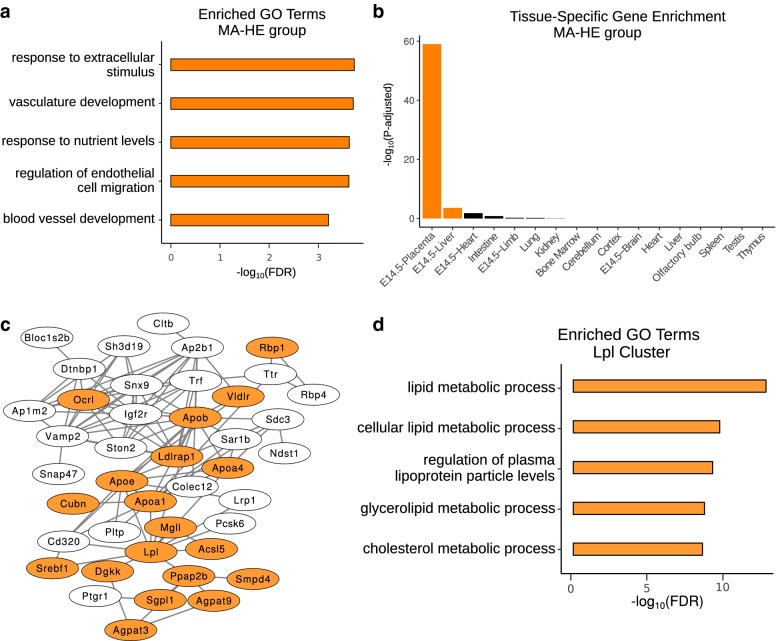
Analysis of MA–HE genes. **a** Bar plot of the enriched GO biological process terms in the MA–HE group, showing terms related to placental functions. **b** Tissue-specific gene enrichment analysis shows enrichment of placenta-specific genes in the MA–HE group. Colored bars correspond to tissues with an adjusted *p* value ≤ 0.01. **c** A network from GLay clustering of MA–HE genes. Orange nodes correspond to genes associated with the GREAT terms shown in **d**. **d** Bar plot of the enriched GO biological process terms in the network from **c** showing enrichment of lipid metabolism terms

A large number of genes (783) were found to have medium–low promoter accessibility and high expression (Fig. 2b; Additional file 1: Figure S4a). GO analysis of these genes showed enrichment for terms associated with placental functions, including “vasculature development” and “response to nutrient levels” (Fig. 4a). In order to determine whether these terms are enriched in the MA–HE group but not the HA–HE group because the HA–HE group is much larger, we performed a randomization analysis in which we sampled 783 genes from the HA–HE group 10,000 times and determined the enrichment of a subset of terms from Fig. 4a. We found that the FDR of these terms is consistently more significant in the MA–HE group than in the random sets from HA–HE, indicating that the large size of the HA–HE group is not masking the signal of these terms (Additional file 1: Figure S4b–d). Since the GO terms were related to, but not specific to, placental functions, we also used TissueEnrich to perform tissue-specific gene enrichment analysis on the MA–HE group, which showed strong enrichment for placenta-specific genes (Fig. 4b).

Nucleosome formation potential has been found to be significantly higher in the 5′ regions of tissue-specific genes compared to housekeeping genes [49]. Therefore, we predicted that MA–HE genes would have higher nucleosome formation potential than HA–HE genes. We randomly chose 250 genes from each group and determined the nucleosome positioning scores using Recon [50]. We found that the MA–ME group had the highest formation potential, followed by the MA–HE group, and that the HA–HE group showed the lowest nucleosome formation potential [Additional file 1: Figure S4e (left)]. To more thoroughly compare the HA–HE and MA–HE groups, we repeated the analysis using the entirety of the two groups and found a similar pattern and, as we predicted, MA–HE genes have significantly higher nucleosome positioning scores than HA–HE genes (*p* value < 2.2E−16) [Additional file 1: Figure S4e (middle)]. This result was further confirmed using nuScore [51] to predict the average deformation energy across all sequences within a group. In agreement with the Recon results, we found that the MA–HE group has a higher deformation energy than the HA–HE group [Additional file 1: Figure S4e (right)].

Because the placenta has many roles and is composed of cell types with diverse functions, we wanted to determine whether the MA–HE group could be further divided into regulatory networks associated with specific placental processes. Using the STRING database, we identified a highly connected network of 318 genes, after which we implemented GLay community clustering [52] through Cytoscape [53]. GLay community clustering is an algorithm used to break up large, dense networks into highly connected clusters of interacting genes. GLay clustering resulted in 10 clusters of at least 10 genes. One such cluster (Fig. 4c) was enriched for terms related to lipid metabolism (Fig. 4d) and contained the lipoprotein lipase (Lpl) gene and several members of the apolipoprotein family including Apob, Apoa1, Apoa4, and Apoe. Many lipoproteins act as transporters for cholesterol throughout the body and across the placenta [54]. Lpl is mainly expressed in syncytiotrophoblast in humans [55] and decomposes lipoproteins into fatty acids. Interestingly, overexpression of Lpl affects nutrient transport and may result in severe intrauterine growth restriction [56]. In another cluster, Trp53, a gene which encodes the tumor-suppressing p53 protein and has been found to be increased in preeclamptic placentas [57], was the hub node (Additional file 1: Figure S4f). Many insulin-like growth factor-binding proteins were also in this cluster, such as Igfbp4 and Igfbp5, which have been associated with preeclampsia and extra-villous trophoblast invasion [58]. These results demonstrate that integrating ATAC-seq data and RNA-seq data in the placenta can lead to the identification of distinct networks associated with different placental functions. These networks could be used to identify novel genes involved in the functions.

### Groups defined by accessibility and expression have consistent functions in multiple cell types

**Fig. 5:**
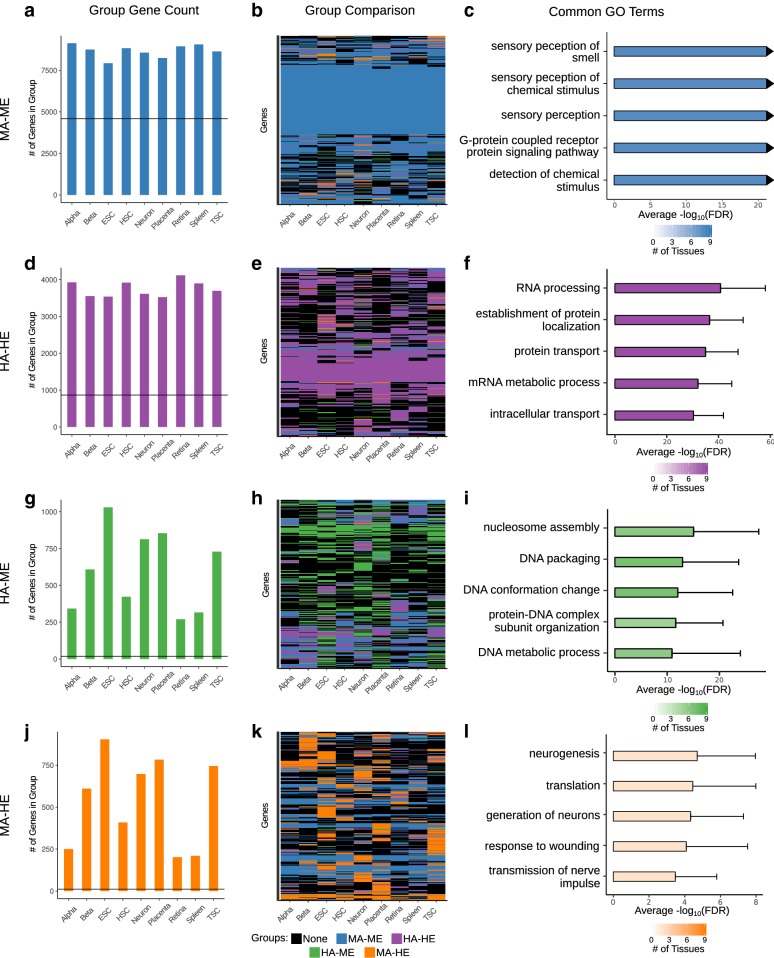
Integrating accessibility and expression data identifies tissue-specific genes in multiple cell types. **a**, **d**, **g**, **j** Bar plots showing the number of genes in each group for all tissues. The horizontal line marks the number of common genes between all tissues for each group, MA–ME (**a**), HA–HE (**d**), HA–ME (**g**), or MA–HE (**j**). **b**, **e**, **h**, **k** Heatmap showing all genes of a particular group for all tissues as well as their corresponding group in other tissues. **c**, **f**, **i**, **l** The top 20 GO terms for each group in each tissue were identified. Then, the number of tissues each term appears in was calculated. The FDR of the term in all tissues in which it appeared was averaged. The terms contained in the top 20 of the most tissues and with the lowest FDR were plotted. The average FDR is shown, and the number of tissues the term appears in is indicated by the intensity of the bar color

To determine whether the functions we identified for the accessibility/expression groups can be generalized to other cell types, we ran our analysis pipeline on data from eight other mouse cell types: alpha cells [59], beta cells [59], embryonic stem cells (ESC) [60, 61], hematopoietic stem cells (HSC) [62], neuronal cells extracted from the dentate gyrus [63], retinal rods [64], lymphocytes from the spleen [65], and trophoblast stem cells (TSC) [66, 67]. First, we checked the correlation between the ATAC-seq promoter accessibility and RNA-seq TPM, which ranged from 0.658 to 0.799 (Additional file 1: Figure S5). Based on the results from the placenta data, we predicted that sensory perception genes would be enriched in the MA–ME group in tissues and cells not associated with such functions and that genes important for general cellular functionality would be enriched in the HA–HE group of all of the cell types. Because of this, we expected that the MA–ME and HA–HE groups would have the highest number of common genes between the datasets. We compared the MA–ME genes between all nine of the datasets (including e9.5 placenta) and found a high degree of gene overlap, with a median pairwise overlap value of 83.5% (7295 genes) (Fig. 5a, b; Additional file 1: Figure S6). Further, the top 20 enriched GO biological process terms for each dataset are similar and related to “sensory perception” and “G-protein coupled receptor protein signaling pathway” (Fig. 5c). A similar analysis of HA–HE genes again showed high gene overlap between all datasets, with a median pairwise overlap value of 63.4% (2374.5 genes) (Fig. 5d, e; Additional file 1: Figure S6). For HA–HE genes, the top 20 GO biological process terms for each set are similar and related to “RNA processing” and “protein transport” (Fig. 5f).

For the HA–ME genes, the median pairwise overlap across all datasets was only 33.7% (177 genes), which is significantly lower than MA–ME genes (*p* value = 2.28e−13) and HA–HE genes (*p* value = 4.335e−12) (Fig. 5g, h; Additional file 1: Figure S6). However, some of the datasets are enriched for terms related to “DNA packaging” (Fig. 5i). Interestingly, in addition to the placenta, only ESC and TSC HA–ME genes showed tissue-specific gene enrichment for brain tissues (cortex, cerebellum, olfactory bulb, e14.5 brain) (Additional file 1: Figure S7a, b). As before, we used the STRING database to look at the protein–protein interaction networks within the HA–ME group from ESC and TSC. For ESC genes, we identified a large subnetwork of 121 genes, enriched for neuron related terms such as “cell communication” and “signal transduction” (Additional file 1: Figure S7c, d). A smaller subnetwork, comprised of 22 genes, was found in TSC HA–ME genes and was also enriched for signaling and neurotransmitter terms (Additional file 1: Figure S7e, f). In addition to the subnetworks enriched for neuron associated functions, we found that Rest/Nrsf is as highly expressed in placenta and TSC as it is in ESC (Additional file 1: Figure S7g). While Rest/Nrsf has been well characterized in ESC, where it prevents neuronal differentiation by repressing neuronal genes in order to promote stem cell pluripotency [68–70], its role in trophoblast cells and placenta is not well understood. Based on these results, we predict that similar repression mechanisms are shared in early placental cells and ESC in order to prevent these cells from adopting a neuronal cell fate.

We next compared the MA–HE genes between datasets to determine whether genes with medium–low accessibility and high expression are always enriched for tissue-specific genes. For these genes, the median pairwise tissue overlap was only 21.5% (88 genes) which is significantly lower than MA–ME genes (*p* value = 2.815e−13) and HA–HE genes (*p* value = 2.381e−12) (Fig. 5j; Additional file 1: Figure S6). Interestingly, of the 2836 genes that are MA–HE in at least one tissue, 1727 (~ 61%) are MA–ME in at least one other tissue, indicating that tissue-specific genes have low accessibility and expression in other tissues (Fig. 5k). We next determined which GO biological process terms and tissue-specific genes were enriched in the MA–HE group of each tissue. The low similarity between the biological process terms (Fig. 5l) and the high enrichment of tissue-specific genes in many of the tissues (Additional file 1: Figure S8) support our previous result in the placenta, indicating that tissue-specific genes and processes are frequently enriched in the MA–HE group.

To determine whether our findings uphold when analyzing human data, we repeated the analysis described above using primary human alpha and beta cells [24]. As with the other datasets, we found a positive correlation between the ATAC-seq promoter accessibility and RNA-seq TPM for both the alpha cells (0.636) and the beta cells (0.616). Similar to what we found in mouse, the MA–ME groups were enriched for sensory perception terms (Additional file 1: Figure S9a, b), and the HA–HE groups were enriched for housekeeping functions (Additional file 1: Figure S9c, d). Finally, we found that MA–HE genes from both cell types were enriched for pancreas-specific genes (Additional file 1: Figure S9e, f), further supporting the conclusions that tissue-specific genes can be identified using this approach.

## Discussion

In this study, we generated ATAC-seq data in the mouse placenta at e9.5, a time point shortly after branching morphogenesis has been initiated, when the vasculature is developing and nutrient transport is active. In order to identify regulatory networks that activate or repress gene transcription at this time point, we integrated ATAC-seq data with RNA-seq data that were generated in the same context. Because previous studies have reported unexpected patterns with respect to promoter accessibility and the corresponding level of gene expression, we investigated this relationship more thoroughly in the placenta as well as in other tissues.

In all tissues, we found a strong positive correlation between the promoter accessibility of a gene and its expression. Although there was a large set of genes contributing to that correlation, which showed consistent expression and accessibility patterns across the tissues, there were other genes that showed opposite patterns of promoter accessibility and expression. To further investigate this, we assigned genes to groups according to their expression and accessibility. We found that MA–ME genes, which have medium–low accessibility and medium–low expression, were enriched for sensory perception terms, not only in the placenta but in all of the cell types we analyzed. Genes with high accessibility and expression were enriched for terms important for general cellular functionality. Previous work has also found that genes with high accessibility in the promoter and high expression are frequently associated with housekeeping functions [27].

Despite the positive correlation between promoter accessibility and gene expression, many studies have indicated that low or decreasing expression is not always due to a lack of accessibility [18, 65, 66]. Others have also found an enrichment of ATAC-seq peaks not only in active regions, but in repressed regions, such as those marked by H3K27me3 [23, 24]. When we investigated genes with high accessibility and medium–low expression (HA–ME), we identified a neuronal network in the placenta, ESC, and TSC, potentially repressed by Rest/Nrsf. Rest/Nrsf is a well-studied transcription factor which, in addition to repressing neuronal gene expression, has also been described to protect against neurodegeneration [71]. In embryonic stem cells, Rest/Nrsf has been found to help maintain pluripotency [70]. Using our pipeline, we were able to identify genes that are potential targets of Rest/Nrsf repression as well as validate H3K27me3 activity within the promoter of nine of these targets. Future work could focus on the identification and validation of Rest/Nrsf targets in the placenta.

While we also identified additional transcription factor motifs that were enriched in promoters of HA–ME genes or other gene groups, the true identity of the factors binding to these motifs would need to be confirmed with follow-up experiments, as many transcription factors have similar motifs. In general, ChIP-seq experiments for transcription factors identified in our analysis would allow in vivo identification of binding sites in gene promoters, as well as in distal regulatory regions, providing more insight into the mechanisms of gene regulation.

Perhaps one of the most surprising results was that by integrating the level of accessibility at the promoter with the level of gene expression, we are able to distinguish between genes associated with housekeeping functions and genes associated with tissue-specific functions. Ontology enrichment analysis and tissue-specific gene enrichment analysis indicate that many genes within the MA–HE group are important for placental functions. There are multiple reasons that highly expressed genes could have medium–low accessibility. One is that tissue-specific genes have higher nucleosome forming potential than housekeeping genes, because they are more selectively accessible, as has been previously proposed [50]. This would likely result in lower ATAC-seq signal. To test this, we used Recon [50] and nuScore [51] and indeed found that MA–HE genes have higher nucleosome forming potential and greater deformation energy in the promoter than HA–HE genes. A second possibility is that the ATAC-seq signal may be low due to the signal only being present in a subset of cells in a heterogeneous tissue, like the placenta. Therefore, we also investigated more homogeneous populations of cells, such as dentate gyrus neurons and TSC. These analyses also identified MA–HE genes that are enriched for tissue-specific functions, indicating results are not specific to heterogeneous tissues. A third possibility is that large regulatory complexes binding at certain promoters prevent the transposase from accessing the DNA. Daugherty et al. [23] investigated binding sites of the EOR-1 transcription factor and found it occupying accessible and inaccessible regions. They hypothesized that EOR-1 may bind near nucleosomes, or as part of a larger complex preventing the transposase from accessing the DNA, causing the low signal at the binding regions. It is feasible that such mechanisms could lead to the low signal near the promoter regions of tissue-specific genes. Along similar lines, it is possible that large regulatory complexes formed by enhancer–promoter interactions would make the promoter less accessible to transposase cutting. In general, further research incorporating additional data sets, as well as distal regulatory information, could help our understanding of the observed signal at tissue-specific genes.

## Conclusion

In summary, we described a pipeline that is not only capable of identifying tissue-specific genes, but also networks of genes that may be actively repressed. This pipeline only requires two types of data, ATAC-seq and RNA-seq. Although many studies focus ≥ strong ATAC-Seq peak, here we discover that a weaker signal of accessibility at the promoter may provide information about the gene’s role in a tissue-specific function. By applying this pipeline to the placenta at one time point, we were able to identify networks of genes associated with specific placental functions, as well as a potential actively repressed neuronal network. We also demonstrated that this analysis can be used in multiple tissues and cell types, in both mouse and human, to similarly identify actively repressed and tissue-specific genes.

## Methods

### ATAC-seq library preparation and sequencing

ATAC-seq was performed as previously described [72]. Briefly, two e9.5 placentas were microdissected from timed-pregnant CD-1 mice (Charles Rivers Labs) for each biological replicate (3 replicates in total). After tissue homogenization and cell lysis, we followed the transposition and purification steps as described previously [72]. We amplified the purified DNA for 12 cycles following conditions specified in [72] using adapters from the Nextera index kit (Illumina) (Additional file 2: Table S3). PCR purification was performed using AMpure XP beads (Beckman Coulter) in order to remove large fragments and remaining primers. Library quality was assessed using the Bioanalyzer High Sensitivity DNA Analysis kit (Agilent) to check for proper periodicity, and the DNA concentrations were estimated using the Qubit and Bioanalyzer (Additional file 1: Figure S1a). Libraries were sequenced by Elim Biopharmaceuticals, Inc. using the Illumina HiSeq2500 Rapid run platform with 50 bp paired-end sequencing. Before filtering, there was an average of about 82 million reads (73–93 million), and after filtering there was an average of about 18.8 million reads (16–20 million) (Additional file 2: Table S4).

### Mouse data curation

Mouse data were compiled from previous studies published until Dec. 2017. Mouse data were considered for analysis if they had at least two biological replicates and had RNA-seq data available in the same cell line and tissue. Only paired-end ATAC-seq data were considered.

### ATAC-seq data processing

Additional ATAC-seq data used for analysis were obtained from the Gene Expression Omnibus (GEO) (Additional file 2: Table S5). For all samples, read quality was assessed using FastQC [73]. Trimmomatic [74] (default settings) was used to remove adapters and low-quality base pairs and reads identified by FastQC. Reads for each mouse sample were aligned to the mouse genome (mm9) and reads for human samples were aligned to the human genome (hg19), using Bowtie2 [75] (v.2.3.1; very-sensitive, maxins 2000, no-discordant). Each sample had high overall alignment rates, with an average alignment of 93% for all mouse samples (Additional file 2: Table S5). After alignment, we removed reads with a mapping quality lower than 20, with more than two mismatches, and those mapping to the mitochondrial genome. We also removed duplicate reads using Picard tools v.2.2.4 (http://broadinstitute.github.io/picard) and offset the reads to account for the transposon dimer [76]. Final read counts for all mouse datasets ranged from 4 to 53 million reads (Additional file 2: Table S4).

Fragments that were less than the length of one nucleosome (140 bp—previously described as subnucleosome sized [77]) were considered as aligning to nucleosome-free, or “open regions” of the genome. After confirming replicates were highly correlated with each other, reads aligning to open regions were combined for all replicates. We then determined the maximum number of overlapping reads (scaled by the library size) in the promoter of each gene. Gene promoters were defined as 500 bp up or downstream of a transcription start site (TSS), and TSS coordinates were obtained from the UCSC Genome Browser knownGene (mm9) [78] table. For genes with multiple isoforms, the isoform promoter with the maximum number of overlapping reads was retained for further analysis.

### RNA-seq data processing

RNA-seq datasets were downloaded from GEO (Additional file 2: Table S6). FastQC was used to check the quality of reads and presence of adapters, and Trimmomatic (default settings) was used to remove adapters and low-quality reads and base pairs. Reads for mouse samples were then aligned to the mouse genome (mm9), and reads for the human samples were aligned to the human genome (hg19), using HISAT2 [79] (v.1.0.4; default parameters). Reads aligning to the mitochondrial genome were removed. Transcript abundance was calculated using htseq-count from the HTseq [80] software package. Transcripts per million (TPM) values were calculated as previously described [81], by normalizing the transcript count by the gene length followed by the library size. Values were then averaged across biological replicates for each gene.

### Integration of ATAC-seq and RNA-seq

Genes were defined as highly accessible (HA) if the maximum number of overlapping ATAC-seq reads within their promoter (accessibility) was higher than the 70th percentile of the data, and were defined as having medium–low accessibility (MA) if the coverage was below the 50th percentile. Genes were defined as highly expressed (HE) if their TPM was higher than the 70th percentile of the data, and were defined as having medium–low expression (ME) if the TPM was below the 50th percentile. Each gene was then evaluated to determine whether it could be assigned to one of four groups: MA–ME (medium–low accessibility and expression), HA–HE (high accessibility and expression), HA–ME (high accessibility and medium–low expression), or MA–HE (medium–low accessibility and high expression) based on its accessibility and expression values. Genes that did not fall into one of the four described categories were not used in the analysis, in order to maintain stringent gene groups.

### Gene ontology and network analysis

Gene ontology enrichment analysis was performed using the Genomic Regions Enrichment of Annotations Tool [26] (GREAT) using the GO biological process ontology. Terms were considered significantly enriched if they had at least 5 genes from the input set, a fold of at least 2, and a false discovery rate (FDR) less than 0.05 according to the hypergeometric statistic [26]. For all analyses, the top 5 terms (ranked by FDR) are displayed unless otherwise indicated.

Protein–protein interaction networks were built using the STRING database [43] (v.10.5). The STRING database provides protein–protein associations determined through experimental data, text-mining, and other databases. The database includes direct and indirect interactions. In a STRING network analysis, a line is drawn between two genes if they are predicted to have an interaction with a certain confidence. We used a confidence threshold of 0.70 and the default parameters in order to build protein–protein interaction networks. STRING networks were downloaded and hub nodes were identified using Cytoscape [53] (v.3.5.1) based on the number of connections between nodes. Clustering of subnetworks was performed using the Cytoscape GLay plugin [52] (default parameters).

### Motif enrichment analysis

The library of position weight matrices used to score binding sites in promoters was obtained by curating data from multiple resources described in [35]. We removed motifs with a low information content (< 10) and those corresponding to genes that had low expression in e9.5 placenta (TPM ≤ 5), leaving 681 motifs. Binding sites were predicted using the PRISM phylofootprint approach [35]. This method has been shown to predict transcription factor binding sites with high accuracy, using an excess conservation metric that measures the likelihood for a binding site to be conserved in a particular region [35]. Binding sites that are conserved more strongly than surrounding sequences are favored [35]. Predictions were required to have a match threshold ≥ 0.8 (80% similarity), a significance of ≤ 0.05 (when compared to shuffled motifs predicted in similar sequences), and had to be conserved in the human genome (hg19).

Similar motifs in the library were identified as previously described [82, 83], using a similarity threshold of 0.8. The motif with the highest number of predicted binding sites was kept for further analysis (Additional file 2: Table S1). For each transcription factor motif, a fold was calculated as the ratio of the proportion of promoters containing the motif in the target gene set to the proportion of promoters containing the motif in a background set of genes. A transcription factor motif was considered to be enriched in the gene set if it had a fold ≥ 2 and a *q* value ≤ 0.05. Significance was determined using the Bonferroni-corrected hypergeometric *p* value.

### Tissue-specific gene enrichment analysis

We used TissueEnrich [48] to carry out tissue-specific gene enrichment analysis, using all of the tissue-specific genes from the mouse ENCODE dataset and default parameters. The enrichment was calculated by using the hypergeometric test. Tissue-specific gene sets were considered enriched if they had an adjusted *p* value ≤ 0.01.

### H3K27me3 chromatin immunoprecipitation (ChIP)

Placentas were microdissected from e9.5 timed-pregnant CD-1 mice (Charles Rivers Labs). Three placentas were combined per biological replicate, and three biological replicates were carried out. Chromatin isolation was carried out as previously described in [84] except that the final nuclear lysis was performed in 0.5% of TritonX-100, 1 mM EDTA, 10 mM Tris–Cl, pH 8.1 and the chromatin was sheared using the Bioruptor Pico for a total of 6 cycles (30 s on/30soff). The H3K27me3 ChIP was carried out with 10ug of chromatin and 4ug of H3K27me3 antibody (Millipore 17-622, lot:3070997) as per manufacturer’s recommendation following the ChIP protocol as previously described in [84]. We quantified the level of H3K27me3 activity with Real-Time qPCR. Gapdh and sheared genomic DNA (input DNA) were used for normalization and enrichment calculation, using the ∆∆Ct method. Primer sequences and efficiency values, calculated after testing 1:4 dilutions of input DNA, are listed in Additional file 2: Table S7.

### Randomization analysis

Randomization analysis was performed to determine the significance of specific ontology terms within subnetworks or gene groups. We generated 10,000 gene sets that matched the size of the group or subnetwork of interest. For each of the random sets, we obtained the FDR of a specific term using GREAT. The *p* value is equivalent to the number of random sets that have a lower FDR than our original term, divided by 10,000.

### Comparison of gene groups between tissues

The pairwise comparison of genes in each group for all tissues (Additional file 1: Figure S6) was calculated by comparing the genes of the same group between each pair of tissues. The Mann–Whitney *U* test was used to check whether the set of overlap counts was significantly different between groups.

### Recon and nuScore analysis

For each gene, we created a fasta file for the region 500 bp upstream of the TSS. These sequences were used as input to the Recon (http://wwwmgs.bionet.nsc.ru/mgs/programs/recon/) or the nuScore (http://compbio.med.harvard.edu/nuScore/) web-tools [50, 51], to obtain nucleosome position scores or deformation energy, respectively. NuScore was run using the 2cv5 human template and default parameters. Recon was also run with the default parameters. Nucleosome position scores for each position were averaged across all sequences within a particular group. Similar averaging was also done on the deformation energy across all sequences within a group. To determine whether the mean nucleosome position score was significantly different between the tested groups, we used the Mann–Whitney *U* test.

## Additional files

**Additional file 1.** Includes supplemental figures S1–S9. **Figure S1.** ATAC-seq on whole tissues shows expected periodicity and correlations. **Figure S2.** The HA–HE group contains a large number of housekeeping genes. **Figure S3.** The HA–ME group is marked by H3K27me3 and is associated with neuronal functions. **Figure S4.** The MA–HE group is enriched for tissue-specific genes. **Figure S5.** ATAC-seq and RNA-seq show high correlation in all tested cell types. **Figure S6.** Pairwise comparison of genes in each group for all tissues. **Figure S7.** HA–ME genes in ESC and TSC have potentially repressed neuronal networks. **Figure S8.** Tissue-specific gene expression in MA–HE groups. **Figure S9.** Group analysis of human alpha and beta cells.

**Additional file 2.** Includes the supplemental tables S1–7. **Table S1.** Enriched Binding Motifs. **Table S2.** Enriched Motifs in HA–ME promoters. **Table S3.** Nextera adapters used for ATAC-seq. **Table S4.** Read counts for ATAC-seq data. **Table S5.** Datasets used for ATAC-seq analysis. **Table S6.** Datasets used for RNA-seq analysis. **Table S7.** H3K27me3 primers.

## Abbreviations

ATAC-seq: assay for transposase-accessible chromatin with high-throughput sequencing
ChIP-seq/qPCR: chromatin immunoprecipitation followed by high-throughput sequencing/quantitative PCR
ESC: embryonic stem cells
FDR: false discovery rate
GO: Gene Ontology
GREAT: Genomic Regions Enrichment of Annotations Tool
HA–HE: high accessibility and expression
HA–ME: high accessibility and medium–low expression
HSC: hematopoietic stem cells
MA–HE: medium–low accessibility and high expression
MA–ME: medium–low accessibility and medium–low expression
PPI: protein–protein interaction
TPM: transcripts per million
TSC: trophoblast stem cells
TSS: transcription start site
UCSC: University of California, Santa Cruz
