# Supplementary material for: Combined analysis of dissimilar promoter accessibility and gene expression profiles identifies tissue-specific genes and actively repressed networks
Source: Epigenetics Chromatin. 2019 Feb 22;12:16. doi: 10.1186/s13072-019-0260-2 (PMC6385419; doi:10.1186/s13072-019-0260-2)
Supplement: Supplementary file 1 — Additional file 1. Includes supplemental figures S1–S9. Figure S1. ATAC-seq on whole tissues shows expected periodicity and correlations. Figure S2. The HA–HE group contains a large number of housekeeping genes. Figure S3. The HA–ME group is marked by H3K27me3 and is associated with neuronal functions. Figure S4. The MA–HE group is enriched for tissue-specific genes. Figure S5. ATAC-seq and RNA-seq show high correlation in all tested cell types. Figure S6. Pairwise comparison of genes in each group for all tissues. Figure S7. HA–ME genes in ESC and TSC have potentially repressed neuronal networks. Figure S8. Tissue-specific gene expression in MA–HE groups. Figure S9. Group analysis of human alpha and beta cells. [file 13072_2019_260_MOESM1_ESM.pdf]

TITLE: Combined analysis of dissimilar promoter accessibility and gene expression profiles identifies tissue-specific genes and actively repressed networks

Rebekah R. Starks[1,2], Anilisa Biswas[1,3], Ashish Jain[1,2], Geetu Tuteja[1,2,3,*]

rstarks1@iastate.edu, anilisa@iastate.edu, jain@iastate.edu, geetu@iastate.edu

[1] Genetics, Development, and Cell Biology, Iowa State University, Ames, IA, 50011, USA

[2] Bioinformatics and Computational Biology, Iowa State University, Ames, IA, 50011, USA

[3] Molecular, Cellular, and Developmental Biology, Iowa State University, Ames, IA, 50011, USA

*Corresponding author

Geetu Tuteja: geetu@iastate.edu

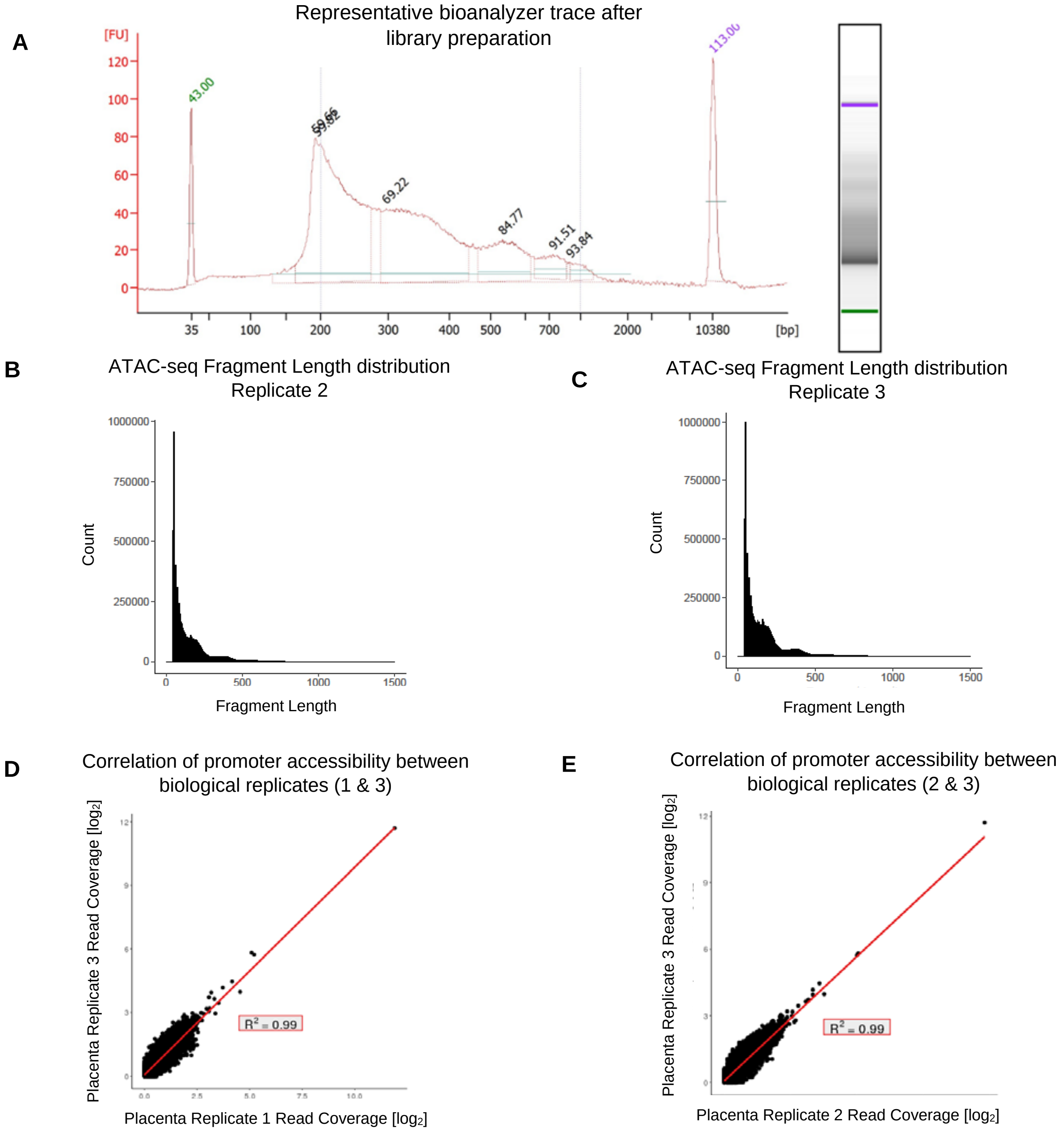

Figure S1. ATAC-seq on whole tissues shows expected periodicity and correlations. (**A**) Representative bioanalyzer trace for an e9.5 ATAC-seq library prior to sequencing. Clear and even periodicity can be seen in the trace. (**B**,**C**) Histogram of fragment sizes for replicate 2 and replicate 3, showing expected periodicity. (**D**,**E**) Scatterplot comparing promoter accessibility between biological replicates. Pearson correlation is high between all replicates.

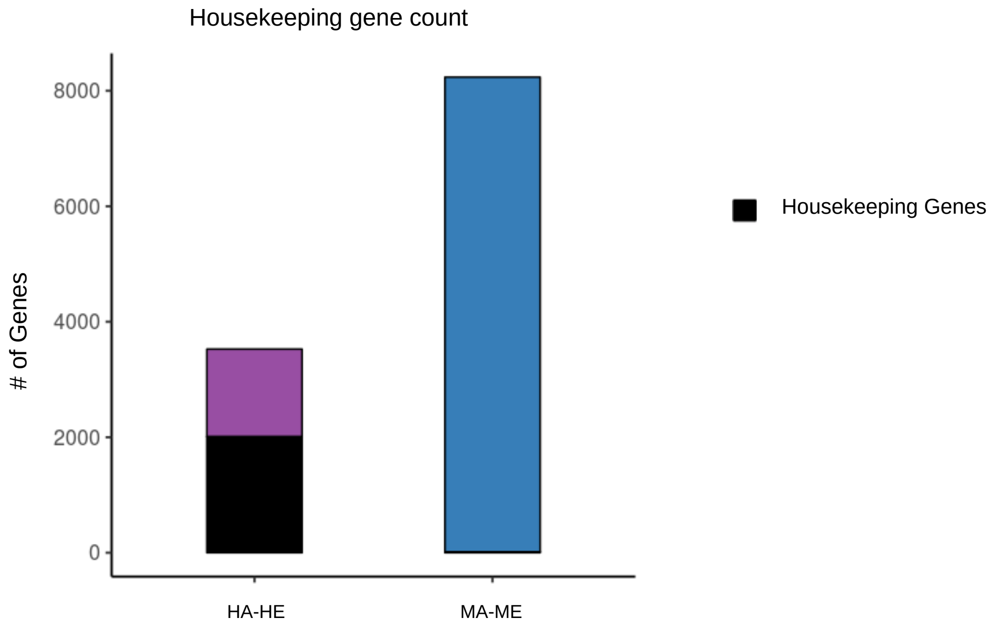

Figure S2. The HA-HE group contains a large number of housekeeping genes. The bar graph shows the number of genes in the MA-ME group and the HA-HE group that are considered housekeeping according to Bin Li et al. (2017) (black bar) and the total number of genes in each group (black bar + colored bar).

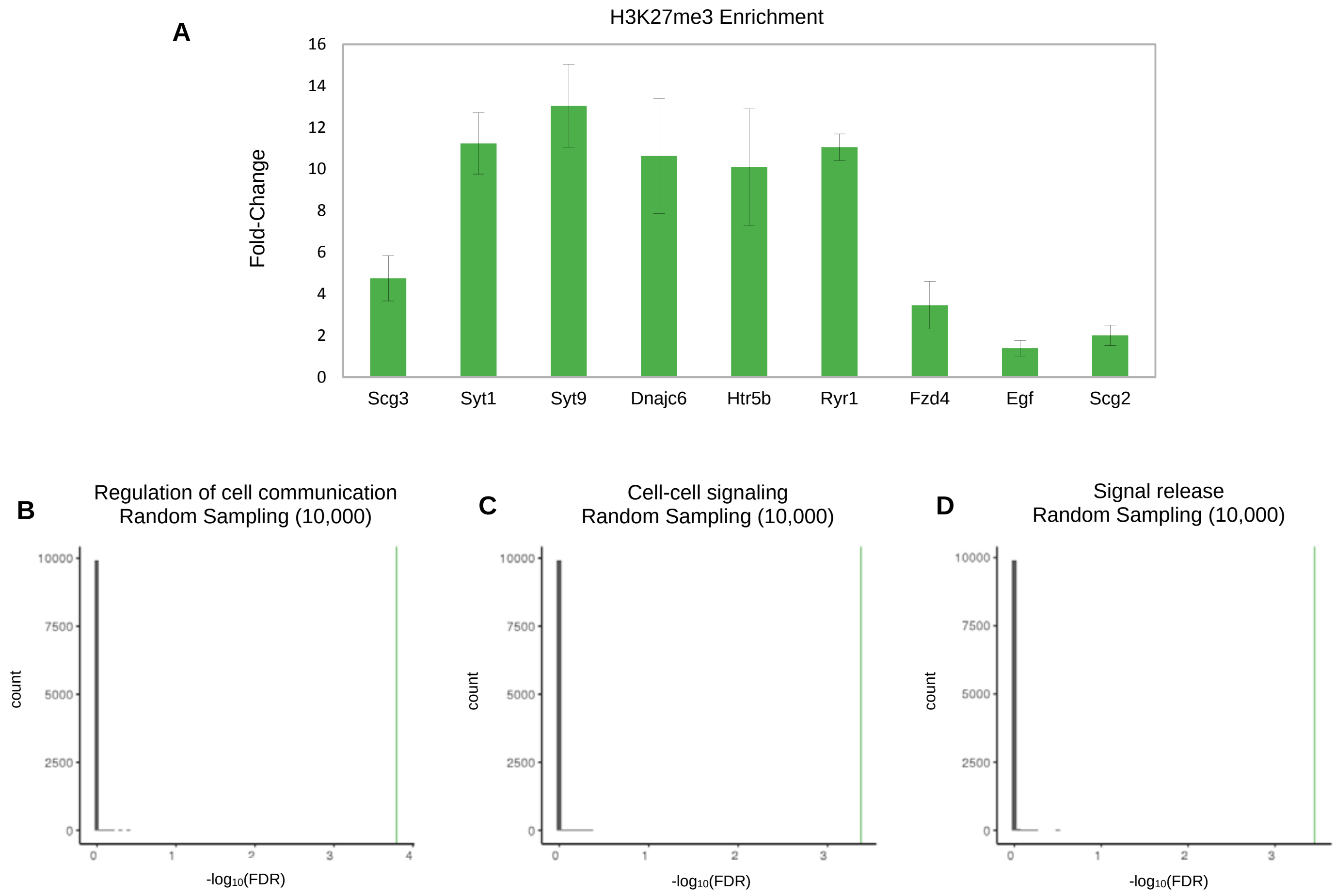

Figure S3. The HA-ME group is marked by H3K27me3 and is associated with neuronal functions. (**A**) H3K27me3 ChIP-enrichment relative to Gapdh in promoters of HA-ME genes. Bar plot shows mean enrichment of the nine tested genes, error bars indicate standard deviation. ChIP was performed for 3 biological replicates. (**B-D**) Bar plots representing the $-\log_{10}$ (FDR) of the specified term from random subgroups of HA-ME genes. The green line denotes the $-\log_{10}$ (FDR) of the term in the subnetwork from Figure 3C in the main manuscript.

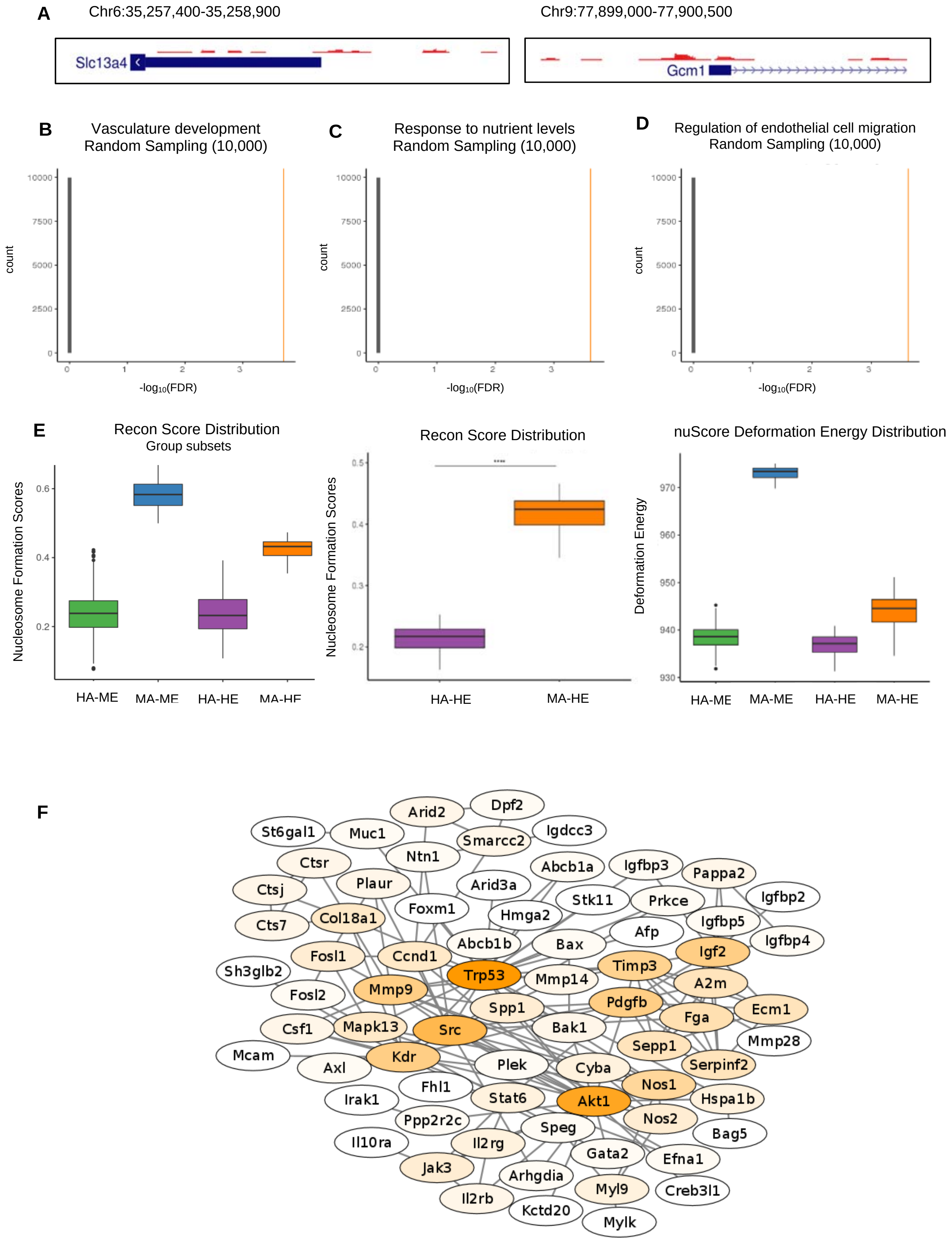
A
Chr6:35,257,400-35,258,900
Slc13a4
Chr9:77,899,000-77,900,500
Gcm1
B
Vasculature development
Random Sampling (10,000)
C
Response to nutrient levels
Random Sampling (10,000)
D
Regulation of endothelial cell migration
Random Sampling (10,000)
count
-log10(FDR)
E
Recon Score Distribution
Group subsets
Nucleosome Formation Scores
HA-ME
MA-ME
HA-HE
MA-HE
Recon Score Distribution
****
nuScore Deformation Energy Distribution
Deformation Energy
F
Arid2
Dpf2
St6gal1
Muc1
Smarcc2
Igdcc3
Ctsr
Ntn1
Abcb1a
Igfbp3
Pappa2
Ctsj
Plaur
Arid3a
Stk11
Prkce
Igfbp2
Col18a1
Foxm1
Hmga2
Igfbp5
Cts7
Afp
Igfbp4
Abcb1b
Bax
Igf2
Fosl1
Ccnd1
Trp53
Timp3
Sh3glb2
Mmp14
A2m
Mmp9
Fosl2
Spp1
Pdgfb
Ecm1
Csf1
Mapk13
Src
Bak1
Fga
Mmp28
Sepp1
Mcam
Kdr
Axl
Plek
Cyba
Serpinf2
Fhl1
Nos1
Irak1
Stat6
Akt1
Hspa1b
Ppp2r2c
Nos2
Speg
Bag5
Il10ra
Il2rg
Gata2
Efna1
Jak3
Arhgdia
Myl9
Creb3l1
Il2rb
Kctd20
Mylk

Figure S4. The MA-HE group is enriched for tissue-specific genes. (**A**) Representative genes from the MA-HE group. ATAC-seq track, displayed in red, shows low accessibility. (**B-D**) Bar plots representing the $–\log_{10}$ (FDR) of the specified term from random subgroups of HA-HE genes. The orange line denotes the $–\log_{10}$ (FDR) of the terms in the MA-HE group. (**E**) Recon score distribution for 250 randomly chosen genes from each of the four groups (left). Recon score distribution for the entire HA-HE and MA-HE groups (middle). MA-HE genes have significantly higher nucleosome positioning scores than HA-HE (middle; **** represents p-value < 0.0001, Mann-Whitney U test). NuScore deformation energy for all of the genes in all of the groups (right). MA-HE genes have a higher deformation energy than HA-HE genes. (**F**) MA-HE gene cluster identified by the GLay community clustering algorithm. Color intensity represents number of edges.

Tissue / Cells	Correlation	Scatterplot
Alpha	0.796	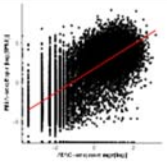
Beta	0.767	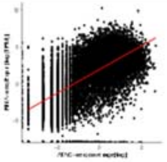
ESC	0.658	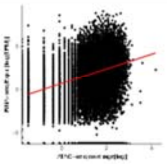
HSC	0.777	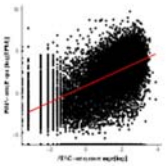
Neuron	0.751	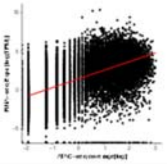
Retina	0.799	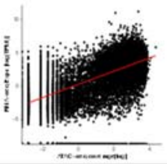
Spleen	0.779	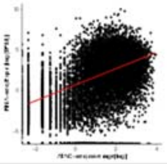
TSC	0.738	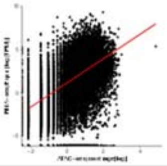

Figure S5. ATAC-seq and RNA-seq show high correlation in all tested cell types. Spearman correlations (column 2) between promoter accessibility and RNA-seq TPM are high in all cell types. The corresponding scatterplot with the linear regression line is shown in column 3.

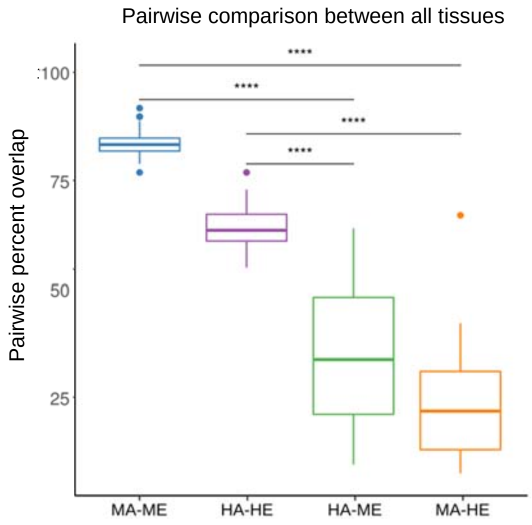

Figure S6. Pairwise comparison of genes in each group for all tissues. Boxplots illustrate the distribution of pairwise percent overlap of genes between all pairs of tissues for each group (**** = p-value < 0.0001, Mann-Whitney U test).

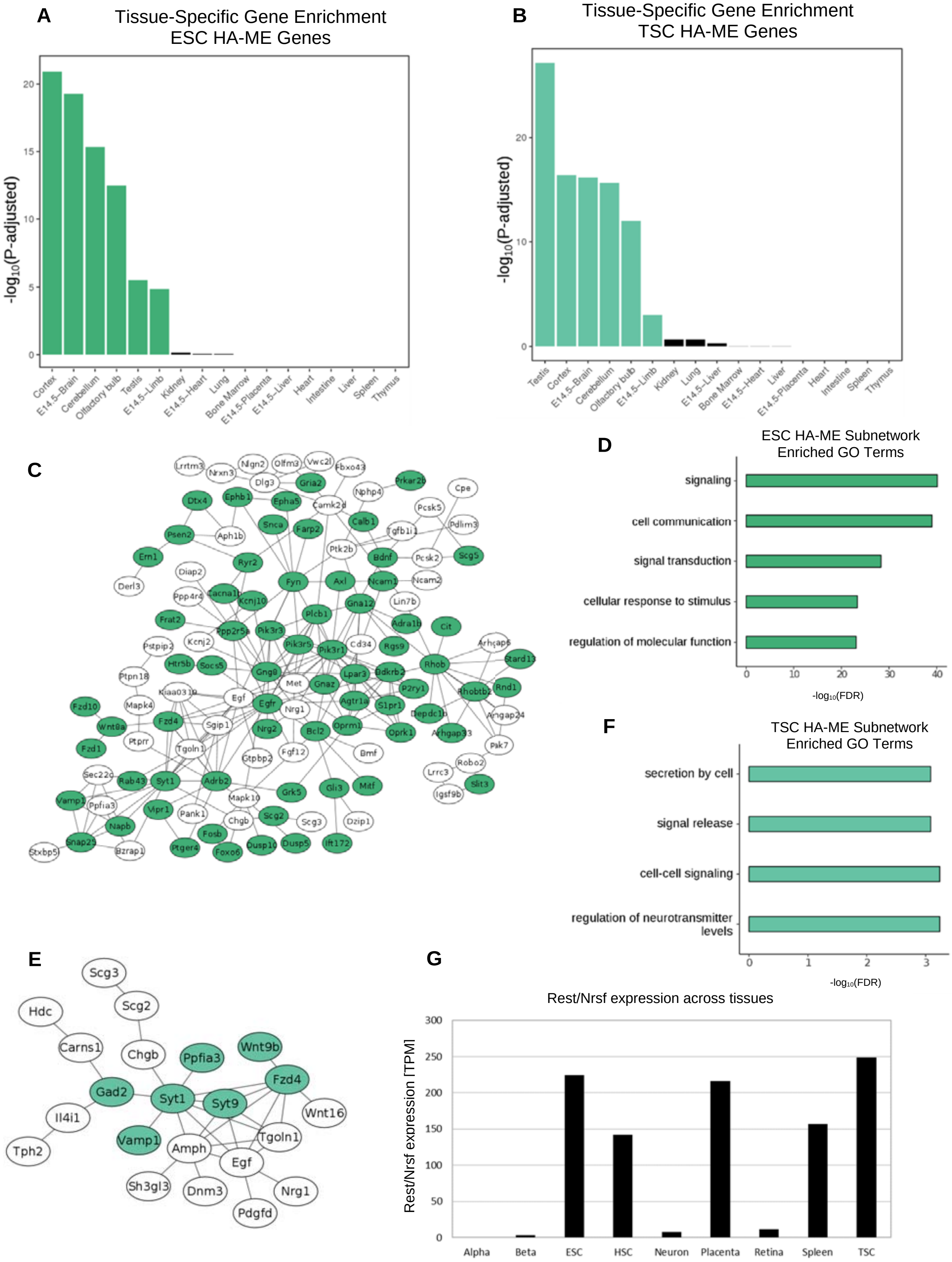

A
Tissue-Specific Gene Enrichment
ESC HA-ME Genes
-log10(P-adjusted)
0
5
10
15
20
Cortex
E14.5-Brain
Cerebellum
Olfactory bulb
Testis
E14.5-Limb
Kidney
E14.5-Heart
Lung
Bone Marrow
E14.5-Placenta
E14.5-Liver
Heart
Intestine
Liver
Spleen
Thymus
B
Tissue-Specific Gene Enrichment
TSC HA-ME Genes
-log10(P-adjusted)
0
10
20
Testis
Cortex
E14.5-Brain
Cerebellum
Olfactory bulb
E14.5-Limb
Kidney
Lung
E14.5-Liver
Bone Marrow
E14.5-Heart
Liver
E14.5-Placenta
Heart
Intestine
Spleen
Thymus
C
D
ESC HA-ME Subnetwork
Enriched GO Terms
signaling
cell communication
signal transduction
cellular response to stimulus
regulation of molecular function
0
10
20
30
40
-log10(FDR)
F
TSC HA-ME Subnetwork
Enriched GO Terms
secretion by cell
signal release
cell-cell signaling
regulation of neurotransmitter levels
0
1
2
3
-log10(FDR)
E
Scg3
Hdc
Scg2
Carns1
Chgb
Ppfia3
Wnt9b
Fzd4
Gad2
Syt1
Syt9
Wnt16
Il4i1
Tgoln1
Vamp1
Amph
Tph2
Egf
Sh3gl3
Dnm3
Nrg1
Pdgfd
G
Rest/Nrsf expression across tissues
Rest/Nrsf expression [TPM]
0
50
100
150
200
250
300
Alpha
Beta
ESC
HSC
Neuron
Placenta
Retina
Spleen
TSC

Figure S7. HA-ME genes in ESC and TSC have potentially repressed neuronal networks. (**A**) Tissue-specific gene enrichment analysis showing that the ESC HA-ME group is enriched for genes specifically expressed in multiple tissues, including the brain (E14.5 brain, olfactory bulb, cerebellum, and cortex). Colored bars correspond to tissues with an adjusted p-value ≤ 0.01. (**B**) Tissue-specific gene enrichment analysis showing that the TSC HA-ME group is enriched for genes specifically expressed in multiple tissues, including the brain (E14.5 brain, olfactory bulb, cerebellum, and cortex). Colored bars correspond to tissues with an adjusted p-value ≤ 0.01. (**C**) PPI network obtained from genes in the ESC HA-ME group. Colored nodes represent genes related to the GO terms in (**D**). (**D**) Bar plot of the enriched GO biological process terms for the subnetwork from (**C**). Enriched terms are related to neuronal functions. (**E**) PPI network obtained from genes in the TSC HA-ME group. Colored nodes represent genes related to the GO terms in (**F**). (**F**) Bar plot of the enriched GO biological process terms, from STRING, for the subnetwork from (**E**). Enriched terms are related to neuronal functions. (**G**) Rest/Nrsf expression in all datasets. Rest/Nrsf expression is high in tissues without neuronal function.

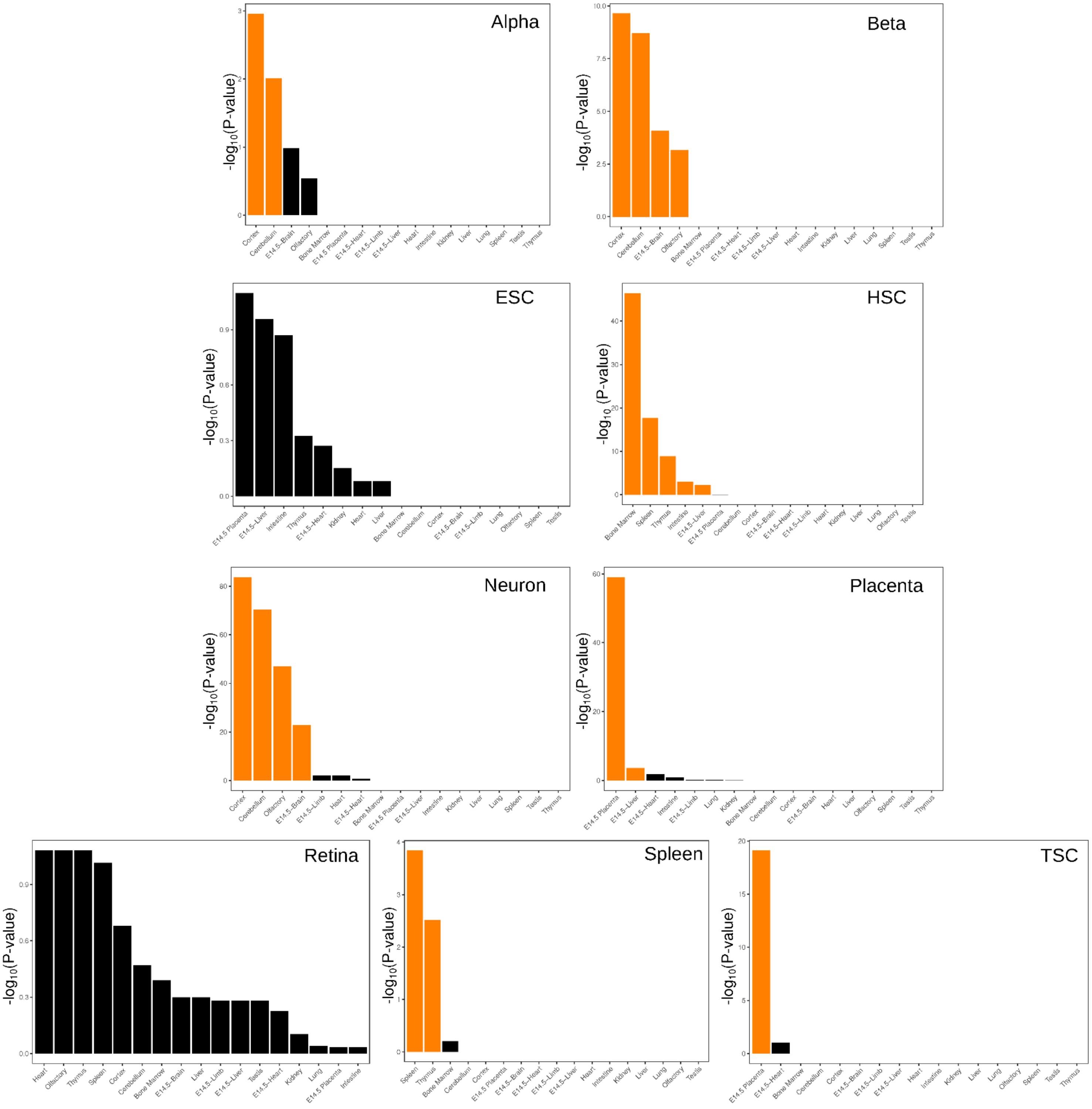

Figure S8. Tissue-specific gene expression in MA-HE groups. Tissue-specific gene enrichment analysis for the MA-HE group of each tissue showing that for most tissues, there is an enrichment of genes specifically expressed in those tissues. Retina and pancreatic cell data are not part of the mouse Encode data, which was used for tissue-specific gene enrichment analysis. Colored bars correspond to tissues with an adjusted p-value ≤ 0.01.

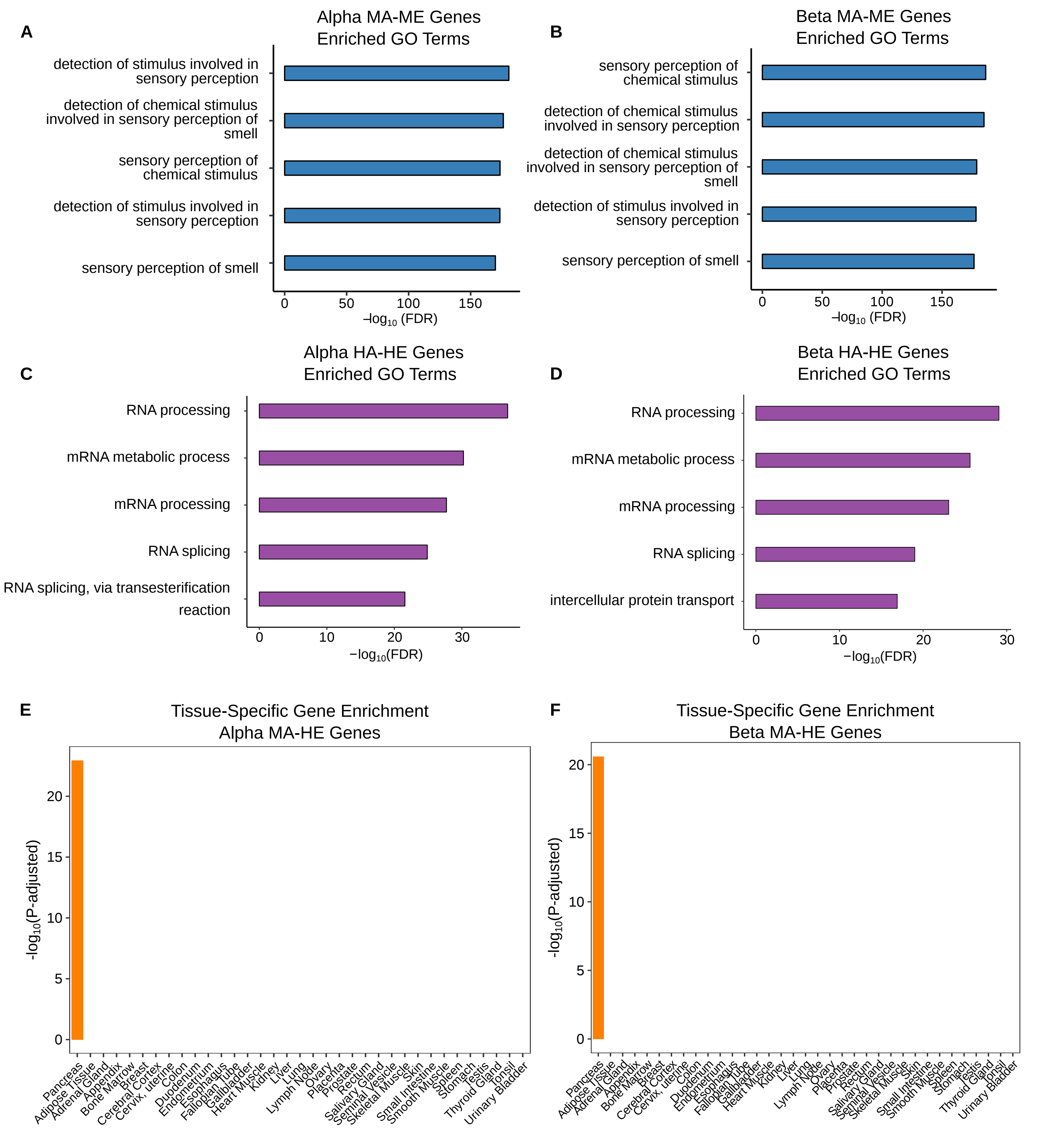
A
Alpha MA-ME Genes
Enriched GO Terms
detection of stimulus involved in sensory perception
detection of chemical stimulus involved in sensory perception of smell
sensory perception of chemical stimulus
detection of stimulus involved in sensory perception
sensory perception of smell
0
50
100
150
−log10 (FDR)
B
Beta MA-ME Genes
Enriched GO Terms
sensory perception of chemical stimulus
detection of chemical stimulus involved in sensory perception
detection of chemical stimulus involved in sensory perception of smell
detection of stimulus involved in sensory perception
sensory perception of smell
0
50
100
150
−log10 (FDR)
C
Alpha HA-HE Genes
Enriched GO Terms
RNA processing
mRNA metabolic process
mRNA processing
RNA splicing
RNA splicing, via transesterification reaction
0
10
20
30
−log10(FDR)
D
Beta HA-HE Genes
Enriched GO Terms
RNA processing
mRNA metabolic process
mRNA processing
RNA splicing
intercellular protein transport
0
10
20
30
−log10(FDR)
E
Tissue-Specific Gene Enrichment
Alpha MA-HE Genes
-log10(P-adjusted)
0
5
10
15
20
Pancreas
Adipose Tissue
Adrenal Gland
Appendix
Bone Marrow
Breast
Cerebral Cortex
Cervix, uterine
Colon
Duodenum
Endometrium
Esophagus
Fallopian Tube
Gallbladder
Heart Muscle
Kidney
Liver
Lung
Lymph Node
Ovary
Placenta
Prostate
Rectum
Salivary Gland
Seminal Vesicle
Skeletal Muscle
Skin
Small Intestine
Smooth Muscle
Spleen
Stomach
Testis
Thyroid Gland
Tonsil
Urinary Bladder
F
Tissue-Specific Gene Enrichment
Beta MA-HE Genes
-log10(P-adjusted)
0
5
10
15
20
Pancreas
Adipose Tissue
Adrenal Gland
Appendix
Bone Marrow
Breast
Cerebral Cortex
Cervix, uterine
Colon
Duodenum
Endometrium
Esophagus
Fallopian Tube
Gallbladder
Heart Muscle
Kidney
Liver
Lung
Lymph Node
Ovary
Placenta
Prostate
Rectum
Salivary Gland
Seminal Vesicle
Skeletal Muscle
Skin
Small Intestine
Smooth Muscle
Spleen
Stomach
Testis
Thyroid Gland
Tonsil
Urinary Bladder

Figure S9. Group analysis of human alpha and beta cells. (**A,B**) Genes in the MA-ME group of human alpha and beta cells are enriched for biological processes related to sensory perception. (**C,D**) Genes in the HA-HE group of human alpha and beta cells are enriched for biological processes related to housekeeping functions. **(E,F)** Tissue-specific gene enrichment analysis shows enrichment of pancreas-specific genes in the MA-HE group for both alpha and beta cells. Colored bars correspond to tissues with an adjusted p-value ≤ 0.01.
